# Bacterial Physiological Adaptations to Contrasting Edaphic Conditions Identified Using Landscape Scale Metagenomics

**DOI:** 10.1128/mBio.00799-17

**Published:** 2017-07-05

**Authors:** Ashish A. Malik, Bruce C. Thomson, Andrew S. Whiteley, Mark Bailey, Robert I. Griffiths

**Affiliations:** a Centre for Ecology and Hydrology, Wallingford, United Kingdom; b The University of Western Australia, Crawley, Australia; Max Planck Institute for Marine Microbiology

**Keywords:** ecophysiology, metagenomics, soil microbiology

## Abstract

Environmental factors relating to soil pH are important regulators of bacterial taxonomic biodiversity, yet it remains unclear if such drivers affect community functional potential. To address this, we applied whole-genome metagenomics to eight geographically distributed soils at opposing ends of a landscape soil pH gradient (where “low-pH” is ~pH 4.3 and “high-pH” is ~pH 8.3) and evaluated functional differences with respect to functionally annotated genes. First, differences in taxonomic and functional diversity between the two pH categories were assessed with respect to alpha diversity (mean sample richness) and gamma diversity (total richness pooled for each pH category). Low-pH soils, also exhibiting higher organic matter and moisture, consistently had lower taxonomic alpha and gamma diversity, but this was not apparent in assessments of functional alpha and gamma diversity. However, coherent changes in the relative abundances of annotated genes between low- and high-pH soils were identified; with strong multivariate clustering of samples according to pH independent of geography. Assessment of indicator genes revealed that the acidic organic-rich soils possessed a greater abundance of cation efflux pumps, C and N direct fixation systems, and fermentation pathways, indicating adaptations to both acidity and anaerobiosis. Conversely, high-pH soils possessed more direct transporter-mediated mechanisms for organic C and N substrate acquisition. These findings highlight the distinctive physiological adaptations required for bacteria to survive in soils of various nutrient availability and edaphic conditions and more generally indicate that bacterial functional versatility with respect to functional gene annotations may not be constrained by taxonomy.

## IMPORTANCE

Over a set of soil samples spanning Britain, the widely reported reductions in bacterial taxonomic richness at low pH were found not to be accompanied by significant reductions in the richness of functional genes. However, consistent changes in the abundance of related functional genes were observed, characteristic of differential ecological and nutrient acquisition strategies between high-pH mineral soils and low-pH organic anaerobic soils. Our assessment at opposing ends of a soil gradient encapsulates the limits of functional diversity in temperate climates and identifies key pathways that may serve as indicators for soil element cycling and C storage processes in other soil systems. To this end, we make available a data set identifying functional indicators of the different soils; as well as raw sequences, which given the geographic scale of our sampling should be of value in future studies assessing novel genetic diversity of a wide range of soil functional attributes.

## INTRODUCTION

Understanding the key drivers and distributions of microbial biodiversity from both taxonomic and functional perspectives is critical to understand element cycling processes under different land management and geo-climatic scenarios. Distributed soil surveys have shown strong effects of soil properties on the taxonomic biodiversity of bacterial communities (1–5) and to a lesser degree for other soil microbes, such as the fungi and protozoa (6, 7). Particularly for bacteria, soil pH often appears as a strong single correlate of biodiversity patterns. This is either due to the direct effects of acidity or a confounding phenomenon whereby soil pH represents a proxy for a variety of other factors across soil environmental gradients. Acidic soils generally harbor reduced phylogenetic diversity and are usually dominated by acidophilic acidobacterial lineages. Intermediate-pH soils (pH 5 to 7) are generally composed of larger numbers of taxa, often with a few dominant lineages, whereas at neutral pH (typically intensive agricultural soils), a more even distribution of a multitude of taxa is typically observed (1, 2, 8). A fundamental issue to be resolved is whether these differences in taxonomic diversity reflect any changes in functional genetic potential. Many dominating organisms, particularly in the more oligotrophic habitats are difficult to culture, and so we know little of the functional characteristics of these organisms at either the phenotypic or genomic level. These knowledge gaps ultimately limit the utility of taxonomic methods (e.g., rRNA based) for further understanding ecosystem function.

Whole-genome shotgun sequencing has been applied to elucidate the functional diversity of communities from a range of ecosystems (8–12), and its application potentially provides novel insights into the genetic diversity of biochemical processes occurring in soils as well as permitting ecological investigations of microbes within a functional trait-based context. The established relationships between edaphic conditions and taxonomic biodiversity can now be complemented with a concurrent understanding of altered environmental microbial physiology and metabolism, which can provide better understanding of the cycling of elements in soils. In the first instance, we need to identify the range of functional genes present in soils to better elucidate the genetic determinants of relevant environmental processes (for example, determining the dominant biochemical pathways most likely to be contributing to fluxes). Second, we need to identify the ecological mechanisms determining how genetic pathways are altered according to environmental change. Addressing these knowledge gaps leads to an understanding of how bacterial diversity in the environment relates to functional potential and importantly how environmental change can impair or enhance soil functionality.

**FIG 1:**
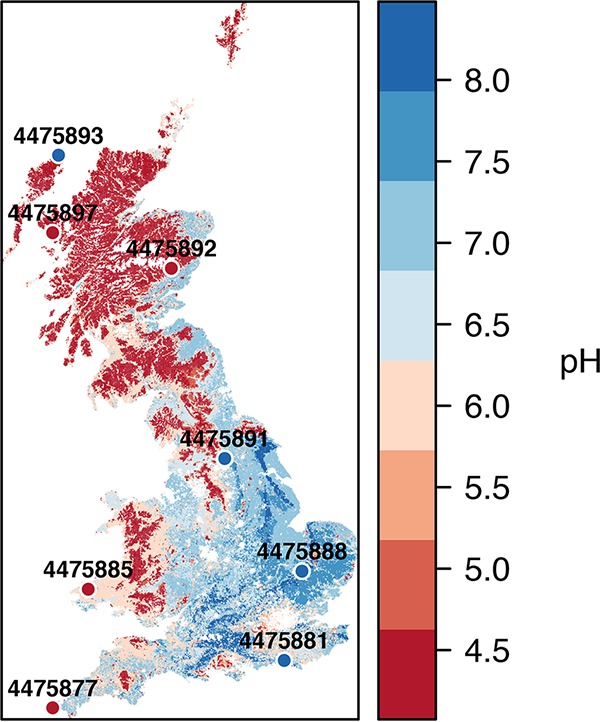
Geographically distributed soils from a range of habitats sampled at opposing ends of a landscape pH gradient. The sampling locations are displayed over a soil pH map of Britain derived from the United Kingdom Soils portal (www.ukso.org).

Here we seek to test whether known differences in taxonomic diversity and composition are reflected in functional gene profiles, by implementing metagenomic sequencing of geographically dispersed soils at opposing ends of a temperate bacterial diversity gradient. We chose to sequence 4 low- and 4 high-pH soils that had previously been collected as part of a national survey of Britain (Fig. 1) and were known to comprise low and high taxonomic diversity, respectively (7). In addition to assessing richness effects, we seek to explore change in specific functional gene abundances in order to elucidate the physiological constraints acting on different soil systems and to identify variance in functional pathways of relevance to soil biogeochemical cycling.

## RESULTS AND DISCUSSION

### Differences in taxonomic richness are not reflected in functional richness.

Four geographically distributed soils encompassing a variety of different habitats but exhibiting similar pHs were selected to represent each of the low- and high-pH classes based on our previous work (Materials and Methods) (Table 1). We note that a variety of other factors covary with pH, with the low-pH soils typically being found at higher altitude and possessing higher organic matter and moisture content consistent with broader patterns across Britain. This association is globally widespread, although it is established that organic matter content itself is not as good a predictor of bacterial diversity as pH (1, 2). For simplicity, we will generally refer to the soils by their pH class while acknowledging the differences in other properties such as organic matter and moisture.

**TABLE 1:** Summary of soil and metagenomic characteristics

Sample ID no.^a^	pH	Land use type	Altitude (m)	Organic matter (%)	Moisture (%)	No. of reads	GC content (%)
4475877	4.5	Heath	215	21.2	56	400,965	60
4475885	4.3	Acid grassland	390	70.4	82	612,521	62
4475892	4.1	Acid grassland	555	47.8	72	647,598	61
4475897	4.4	Coniferous woodland	130	88.8	90	645,976	59
4475881	8.3	Hedgerow	135	24.1	48	556,279	63
4475888	8.4	Improved grassland	75	6.4	25	621,879	63
4475891	8	Arable	70	5.1	21	679,130	64
4475893	8.5	Improved grassland	10	8.9	33	722,025	63

a Functional annotations are available on MG-RAST under the sample ID numbers shown.

**FIG 2:**
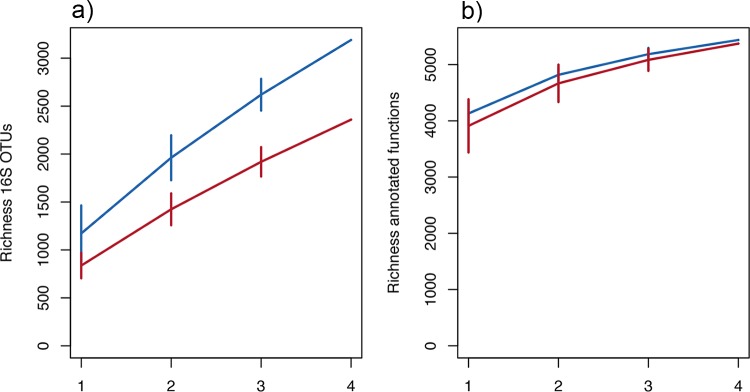
Within-site and across-site taxonomic and functional richness represented by site-based accumulation curves. Standard deviations are calculated from random permutations of the data, with red lines representing low pH and blue lines high pH. (a) Taxonomic richness determined by 16S rRNA sequencing is higher at high pH, both within individual sites (alpha diversity) and accumulated across sites (gamma diversity). (b) Richness of annotated functional genes following whole-genome sequencing is only marginally lower in low pH at individual sites and converges across all sites.

Amplicon sequencing confirmed that the microbial taxonomic richness differed between the two soil pH groups, with both alpha diversity (richness) and gamma diversity (sum of taxonomic richness across all 4 sites) being higher in the high-pH soil communities (Fig. 2a). We then sought to examine whether the richness of annotated genes from metagenomic sequencing was also less in low-pH soils. Metagenomic sequencing using the Roche 454 platform resulted in a total of 4.9 million quality filtered reads across all samples; with 0.4 to 0.7 million reads per sample (Table 1). An average of 8.3% ± 0.4% of sequences failed to pass the Metagenomics Rapid Annotation using Subsystems Technology (MG-RAST) quality control (QC) pipeline. Following gene annotation by MG-RAST using default stringency criteria, the percentage of reads annotated to predicted proteins ranged from 63.3 to 73.7% across samples (average of 68.9%). The majority of annotations were assigned to bacterial taxa (94.7% ± 1.6% in low-pH soils, 96.6% ± 0.3% in high-pH soils), with only a small proportion being eukaryotic (3% ± 1.2% in low-pH soils, 1.8% ± 0.1% in high-pH soils) and archaeal (2.2% ± 1.4% in low-pH soils, 1.4% ± 0.3% in high-pH soils). The marked low proportion of fungi contributing to the soil DNA pool was also reflected in the RDP annotation of rRNA reads (not shown). The metagenomic percentage of GC content was lower in acidic anaerobic soils (Table 1), corroborating previous evidence suggesting a link between aerobiosis and percentage of GC content in prokaryotes (13).

Mean richnesses (alpha diversity) of functionally annotated genes were 4,153 and 3,915 in high- and low-pH soils, respectively (Fig. 2b), although this difference was not statistically significant (*t* test, *P* = 0.12). This was in contrast to the amplicon data, where richness was consistently higher across all samples at high pH (*t* test, *P* < 0.05). No correlative associations were observed between functional and taxonomic richness, in contrast to one previous study across a range of prairie grasslands (8). Importantly however, we found that while the accumulation of taxon richness over sites accentuated the differences in taxonomic diversity (Fig. 2a), this was not true for functional richness, where 4 low-pH soils possessed equivalent total functional gamma diversity to 4 high-pH soils (Fig. 2b). Thus, it is clear that while taxonomic diversity may be restricted by low-pH-related factors, functional diversity of gene categories across multiple samples can be maintained through higher between-site variance (beta diversity), possibly mediated through enhanced metabolic versatility within acidophilic taxa. This clearly needs to be explored further with respect to local variance across smaller spatial scales and may be highly relevant for assessing the effect of local land management contrasts on the spatial structuring of soil functional capacity. Due to our limited selection of sites, we cannot address this here, but future work facilitated by reductions in sequencing costs should focus on more spatially resolved investigations of the role of environmental and spatial variables in determining soil bacterial functional diversity patterns, as routinely conducted for taxonomic diversity assessments (1, 2). We also note that functionally annotated genes appear to be fairly conserved across different soils, although there is undoubtedly considerable genetic variation in the genes coding for these proteins across soils. Future studies employing greater sequencing depth or targeted gene sequencing of relevant genes (see below) will permit detailed phylogenetic diversity contrasts within individual protein-coding genes to better explore concepts of redundancy.

### Large differences in relative gene abundance between low- and high-pH soils.

**FIG 3:**
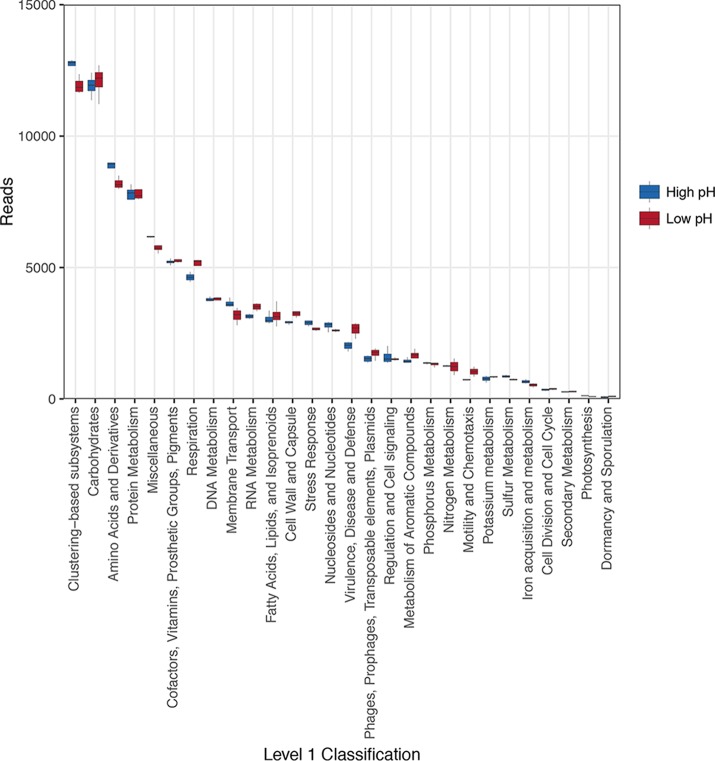
Abundances of annotated functional genes classified at the broadest level (level 1 subsystem classification), with total reads standardized across samples to 92,442 reads.

**FIG 4:**
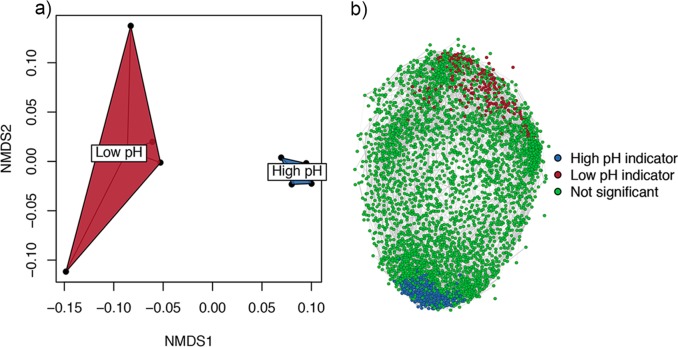
(a) Ordination of functional genes (classified at the level of function) using two-dimensional nonmetric multidimensional scaling (NMDS) reveals strong clustering of sites by pH irrespective of geographical sampling origin. (b) Network depicting strong (>0.9) positive correlations between annotated functional genes. For clarity, rare genes with less than 10 reads across all samples were omitted. Indicator functional genes are colored according to pH class following indicator (IndVal) analyses.

Since a lack of difference in functional richness does not mean that soils are functionally similar across the pH gradient, we next sought to assess differences in relative abundances of functional genes. Figure 3 shows the overall abundance of genes classified at the broadest level (level 1 subsystem classification). Despite similarities in the abundance ranked order of hierarchically classified genes, a number of notable differences between soils of different pHs were immediately apparent for several relevant functional categories, such as amino acid cycling, respiration, membrane transport, stress, and virulence-disease-defense. However, a lack of difference at this level (notable for carbohydrate and nitrogen metabolism) could be due to significant differences within higher-level functional categories. To address this, we next performed a multivariate assessment of gene composition classified at the level of function. This revealed large differences between soils of different pHs, indicating that the pH-defined communities shared more similar functional gene profiles independent of their geographical origin (Fig. 4a; analysis of similarity [ANOSIM], *P* < 0.05). The analyses based on relative abundance therefore show that functional genes were differentially abundant in opposing soil pH classes and, in combination with higher functional beta diversity at low pH, explain the equivalence of alpha diversity metrics.

**FIG 5:**
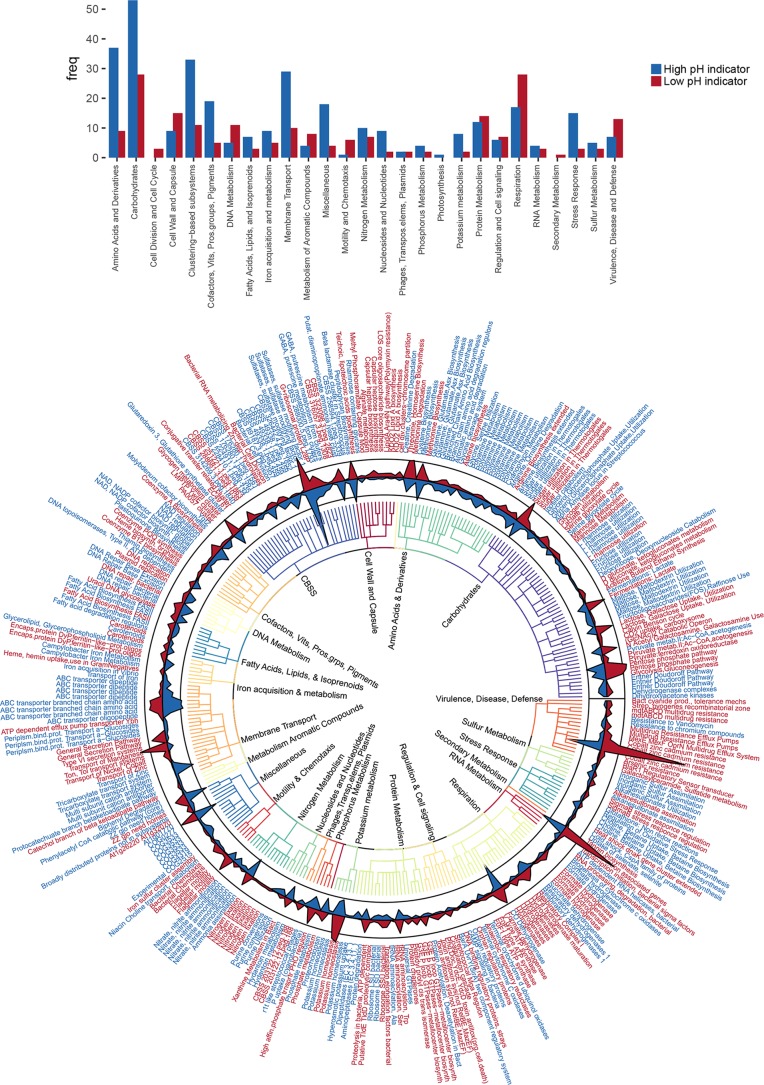
(Top) Bar plot showing the frequency (freq) of indicator genes at the broad level 1 classification. (Bottom) Circular plot displaying the identity and abundances of significant indicator genes for low- and high-pH soils. Nodes represent individual functional indicators, although they are labeled with the more descriptive subsystem level 3 classification (i.e., repeated node labels indicate different functional indicators within the same level 3 subsystem classification). Node labels are colored red and blue for particular genes that are significantly more abundant in low- or high-pH soils, respectively. Line plots represent total abundances of the indicators within the rarefied data sets and are filled according to pH (red, low; blue, high). The tree depicts the hierarchical subsystem classification, with level 1 classifications being labeled on the internal nodes.

Indicator gene analysis (indicator value [IndVal]) was then used to statistically define the characteristic genes contributing to the differences between the low- and high-pH soils. Of a total of 6,194 annotated genes, 206 and 322 significant functional indicators were found for the low- and high-pH soils, respectively. A network depicting only strong positive correlations (>0.9) between genes across all samples revealed an expected lack of connectedness between opposing indicators, and in general low-pH indicator genes were less correlated than high-pH indicators, reflecting the greater magnitude of functional beta diversity across low-pH soils (Fig. 4b). To examine the identity of indicators (significant functional genes only), we constructed a circular plot (Fig. 5) at the functional gene level of classification, omitting rarer genes for ease of presentation (<50 reads across all samples) and labeling the indicators using the respective broader and more descriptive level 3 subsystem classification. A full table of all gene abundances, their classification and indicator statistics is provided in Data Set S1 in the supplemental material. The following subsections discuss some relevant indicators of low- and high-pH soils. While this discussion is by no means exhaustive, we divide the discussion into two sections based on (i) physiological processes for survival and nutrient capture and (ii) metabolism.

10.1128/mBio.00799-17.1 DATA SET S1  Annotated functional gene abundances, their SEED subsystem classification, indicator scores, and *P* values. Refer to Table 1 for unique sample identifiers. Download DATA SET S1, XLSX file, 0.6 MB. Copyright © 2017 Malik et al. 2017 Malik et al. This content is distributed under the terms of the Creative Commons Attribution 4.0 International license.

### **Variable physiological strategies for survival and nutrient acquisition**.

The indicator analysis reveals an array of interlinked microbial physiological adaptations to survival at the extremes of the soil pH spectrum (Fig. 5). First, with respect to cellular physiology, the high-pH soils possessed a greater abundance of ABC transporters relatable to nutrient acquisition (14). In addition to the abundant amino acid, peptide, and tricarboxylate transporter indicators (level 1 classification: membrane transport), numerous other transporters were significantly enriched at high pH, although under different subsystem classifications. These included the majority of carbohydrate indicators for mono- and disaccharide uptake, as well as other transporters for inorganic sulfate, cofactors, polyamines, ammonia/nitrate, potassium uptake proteins, and high-affinity phosphate transporters (15). Interestingly, transporters for iron acquisition as well as osmoprotection were also evident at high pH, possibly reflecting low moisture and iron availability in the selected high-pH soils. Together these findings indicate that the high-pH soils can be distinguished functionally from low-pH soils on the basis of a greater abundance of transporters for the direct uptake of available substrates and cofactors required for growth.

The membrane transport-related indicators of low-pH soils included only two low-abundance genes related to nutrient acquisition (phosphate and glucose [not indicated in Fig. 5 due to low abundance]) but also a number of genes coding for proteins linked to metal acquisition (Ybh, MntH, HoxN/HupN/NixA) and protein secretion (siderophores and extracellular enzymes). Relatedly a number of membrane proteins (mainly proton antiporters) for the efflux of antibiotics and toxic compounds were characteristic of low-pH soils (ACR3, BlaR1/MecR1, CusA, CzcC, MerR, MacB, NodT, MdtB, MdtC), but annotated on the figure under “Virulence, Disease and Defense.” Indeed, the gene for cation efflux protein CusA related to cobalt-cadmium-zinc resistance was one of the more abundant genes across all metagenomes but was significantly enriched in low-pH soils. Given the nature and location of the low-pH soils examined, it is unlikely these represent adaptations to either severe metal contamination or anthropogenic sources of antibiotics, but rather are related to pH-enhanced reactivity of toxicants (e.g., metal ion solubility generally increases with decreasing pH) and the alleviation of acid stress through membrane efflux (16–20). It is also conceivable that they may be required for metal import for a variety of metal-necessitating enzymes (see below) since many are proton/cation antiporters. Supporting this, another strong indicator of low pH was a potassium-transporting ATPase gene (level 3 class: potassium homeostasis) coding for a membrane protein responsible for exchange of H^+^ and K^+^ ions across the plasma membrane, suggesting the coupling of acid stress response with elemental acquisition. It is noteworthy that we were unable to find any studies that have examined acid soils for their potential as a source of antibiotic resistance, but our findings implicate adaptation to acidity or anaerobiosis as a possible factor underlying natural resistance to antibiotics (21, 22). Other notable indicators of low pH included chemotaxis and motility genes, plausible given the higher moisture contents of these soils (18). In total, these results indicate that in the acidic soils under investigation, considerable energy investments must be made in cellular processes to survive in an acid-stressed, oxygen-limited, and low-nutrient environment.

### Metabolic potential of contrasting pH soil communities.

Carbohydrate processing was one of the most abundant broad classes of annotated functional processes, and within this level 2 subsystem class serine-glyoxylate cycle, sugar utilization, and tricarboxylic acid (TCA) cycle genes were identified as the most abundant. Despite the expected conservation of many key metabolic functions, a number of notable indicators were found (Fig. 5). Several genes differed for the processing of mono- and oligosaccharides, with a number of genes for l-rhamnose and fructose utilization being of greater abundance at high pH, whereas several extracellular enzyme-coding genes for glucosidase, β-galactosidase, mannosidase, and hexosamidase were elevated at low pH. With respect to the central carbon metabolism, certain components of the Entner-Doudoroff pathway were reduced in abundance in low-pH soils (gluconate dehydratase, gluconolactonase), but by far the most abundant and significant low-pH indicator was the xylulose 5-phosphate phosphoketolase gene. This gene along with another strong low-pH indicator—the transketolase gene—is part of the pentose phosphate pathway, a metabolic pathway parallel to glycolysis yielding pentose sugars and reducing agents. Although not previously considered largely with respect to soil functionality, this gene was generally of high abundance across all soils. However, novel position-specific isotope tracing experiments have recently provided functional evidence of the significance of the pentose phosphate pathway in soil C cycling (23). We note it is involved in fermentative processes through a variant of the pentose phosphate pathway and therefore would be expected to occur more frequently in these more anaerobic soils (24). Further evidence of an increase in fermentative processes in low-pH soils was seen with a number of indicator genes for pyruvate:ferredoxin oxidoreductase (autotrophic CO_2_ fixation linked to reductive TCA cycle [25]), lactate fermentation, and a wide range of hydrogenases (26, 27). Several recent studies have demonstrated that these hydrogenases are widespread in soil microorganisms (26, 28, 29), and they may also represent another means of consuming protons (30). Among these were cytoplasmic NAD-reducing hydrogenases that may be linked to respiration and fermentation through their NADH generation capabilities (31, 32). The nickel transporters (HoxN/HupN/NixA family) identified earlier may also be linked to this process since nickel is required for the metal center of NiFe hydrogenases (33, 34). Together, the metagenomic data suggest that low-pH soil communities harbor adaptive physiological strategies of using molecular hydrogen oxidation and coupling it with respiration and fermentation to generate energy (35).

While representing only a small proportion of these metagenomes, a number of nitrogen metabolism-associated genes differed significantly between the soils of different pHs. Nitrogen fixation genes and particularly molybdenum-dependent nitrogenases were consistently strong indicators of low-pH soils, as demonstrated by high indicator scores (Data Set S1). Indeed, some of the nitrogenase indicators were unique in being universally present at low pH but entirely absent in the high-pH soils. These findings indicate that microbial N input into soils either through symbiotic or nonsymbiotic routes may be relatively larger in acidic soils, and there was evidence from the taxonomic assignments that the Bradyrhizobia may play a key role in this process (not shown). Low-pH soils are characterized by decreased decomposition rates that could reduce the available N in soils necessitating microbial N fixation (36–38). The coupling of N fixation with abundant hydrogenases may also represent an efficient system for recycling the H_2_ produced in N_2_ fixation, minimizing the loss of energy (33, 39). High-pH soils showed significantly more genes linked to nitrate-nitrite ammonification, ammonia assimilation, and denitrification (Fig. 5). The dominant N cycling pathways appeared to be related to ammonification (nitrate reduction to ammonia) and ammonia assimilation, and these were more abundant in high-pH soils (40). Relatedly, a number of notable indicators of high-pH soils were for the degradation of amino acids and derivatives, such as arginine, ornithine, polyamines, urea, and creatine. Coupled with greater abundance of amino acid and peptide transporters, these observations infer an increased reliance on scavenging N-enriched compounds originating from biotic inputs in high-pH soils (20, 41, 42). Conversely, the lower abundance of these genes at low pH may indicate reduced bioavailability of these compounds, possibly due to its increased adsorption to soil minerals (greater cation exchange capacity at low pH [43]) or simply due to decreased organic matter decomposition in acidic soils.

### Conclusions.

**FIG 6:**
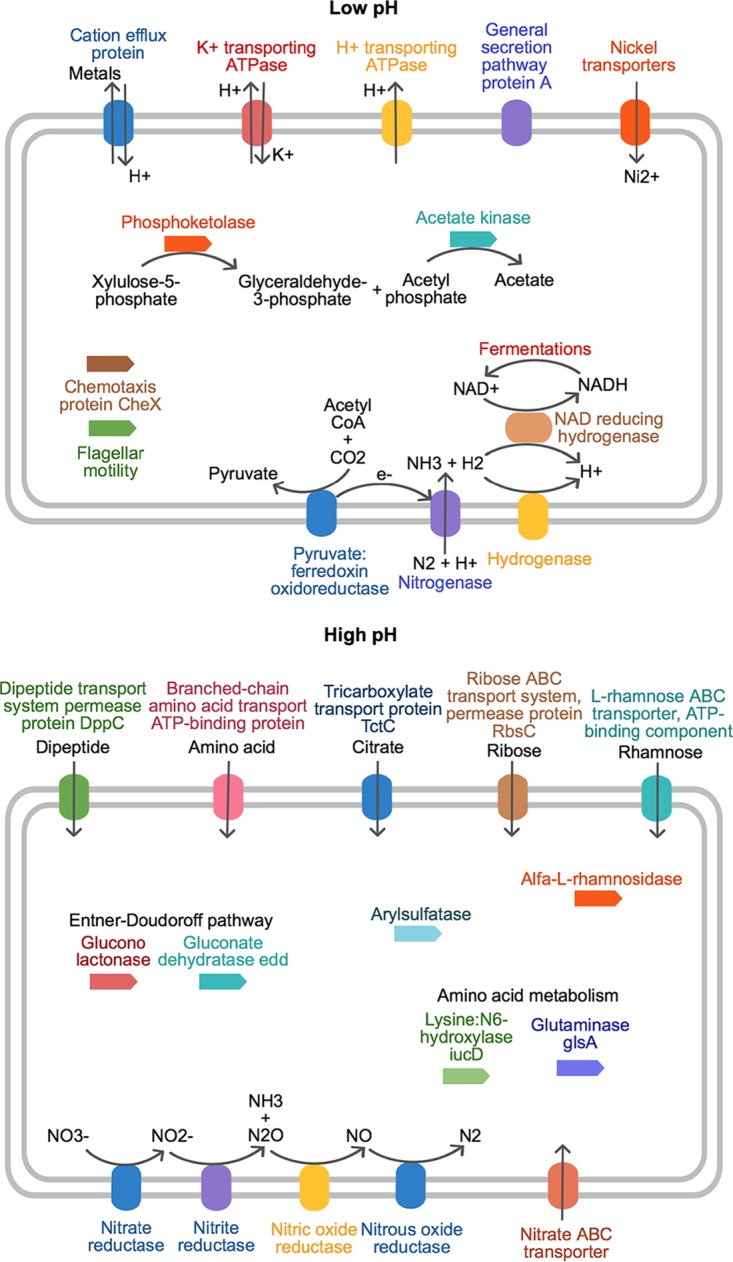
Schematic summarizing some of the main physiological differences for survival, nutrient acquisitions, and substrate metabolism across the pH gradient, as identified from the indicator analyses. We note that inclusion of a gene in either schematic is based on differences in abundances and does not implicate the presence/absence of a particular pathway across the gradient (refer to the circular plot in Fig. 5).

Our study shows that despite large differences in the taxonomic diversity of bacteria known to exist across soil environmental gradients, there was little evidence to suggest this results in large differences in the diversity of functionally annotated genes. Rather, low- and high-pH soils differed in the relative abundance of specific functional genes, and these indicator genes reflected differences in survival and nutrient acquisition strategies caused through adaptation to different environments. For the low-pH soils, there were a number of abundant functional genes that highlight the importance of varied biochemical and physiological processes that we infer to be important adaptations to life in acidic, wet, and oxygen-limited environments. Indeed, our results highlight the coupled action of acidity and anaerobiosis in mediating bacterial functional responses (22). In such soils, a considerable investment of energy must be made in complex processes for capturing nutrients and energy, as well as stress responses caused by both the exterior environment and cellular metabolism. In combination, this is reflected by a greater abundance of cation efflux pumps, C- and N-acquiring systems such as direct fixation, and fermentation (Fig. 6). Higher-pH soils conversely possessed more direct transporter mediated mechanisms for C substrate acquisition together with numerous indicators of organic N acquisition and consequent cycling (Fig. 6).

In identifying specific indicators across the gradient, we acknowledge some limitations to the metagenomic approach. First, the analyses are reliant on accurate functional assignment of reads, and there are recognized issues with respect to both bioinformatic annotation and the experimental assignment of a sequence to a single function (44). For this reason, we have focused our discussion on the more prevalent indicators represented by a variety of individual functional gene categories. An additional concern is that observed changes in relative gene abundance could be simply due to change in an unrelated gene (45). While various methods for standardizing reads have been applied (such as relating abundances to rRNA genes or calculating proportions within discrete subsystems), these approaches are not entirely without scrutiny. We prefer to consider the analyses akin to a mass balance—whereby the relative abundances of genes reflect the proportion of investment made for a given amount of nutrient in different proteins. The relevance for function at the ecosystem scale is a separate line of inquiry necessitating process measurement and assessment of biomass size, and we envisage that metagenomic studies provide more relevant functional targets.

In conclusion, we identify that considerable metabolic diversity and variability can exist within communities of environmentally constrained taxonomic diversity. Our intent at focusing analyses at broad ends of the pH spectrum was inspired to encompass an assessment at the extremes of soil functionality. In doing so, we make available sequence data sets that may be useful to others looking to assess the diversity of specific functional genes for a wide range of soil processes across a range of soils. Furthermore, we envisage that the indicators identified here, while being from an extreme soil contrast, may also be relevant at local scales for understanding more subtle alterations in soil function. For example, more “natural” soils in temperate climates typically store more carbon, tend toward acidity, and have increased moisture retention, whereas human agriculture forces soils to neutrality and depletes soil carbon and moisture. Our results identifying differential nutrient acquisition and alterations in metabolic mechanisms are likely to be more widely relevant in understanding the balance of energy and C storage mechanisms under altered land management, as well as permitting future design of smarter systems for efficient soil nutrient capture and recycling.

## MATERIALS AND METHODS

Eight soils were selected for detailed metagenomic analysis on the basis of soil pH alone and are representative of the extremes of both the soil environmental conditions and bacterial diversity range encountered across Britain. Characteristics of soil examined and location of sampling sites are shown in Table 1 and Fig. 1, and full details of the sampling, nucleic acid extraction, and taxonomic analyses are provided in our previous articles (2, 7). Briefly, samples were taken as part of the Countryside Survey, which comprises an assessment of several hundred 1-km^2^ plots across Britain representing a range of broad land use types. From each of these plots, 5 soil cores (5 cm in diameter, 15 cm deep) were sampled each from randomly allocated subplots. In this study, DNA was extracted from 3 g of soil from a single subplot core at the locations identified in Fig. 1 using a previously described method (46). Bacterial communities were characterized using tagged amplicon sequencing as previously described (7) using primers 28F/519R and sequencing on the 454 pyrosequencing platform through a commercial provider. Raw sequences from amplicon sequencing were analyzed using QIIME, using UCLUST to generate operational taxonomic units (OTUs) at 97% sequence similarity. For whole-genome metagenomic sequencing, 3 μg of total DNA from each sample was submitted to the NERC Biomolecular Analyses Facility (Liverpool, United Kingdom), with two samples per run on the 454 platform (sequencing statistics in Table 1). Resulting sequences from metagenomic analysis were annotated with the Metagenomics Rapid Annotation using Subsystems Technology (MG-RAST) server version 4.0 (47). Functional classification was performed using the SEED Subsystems database with a maximum E value cutoff of 10^−5^, minimum identity cutoff of 60%, and minimum length of sequence alignment of 15 nucleotides. Gene abundance tables derived from MG-RAST were imported into R for downstream analyses.

Rarefaction, species diversity accumulation, ordinations, and statistical analyses were performed using the vegan package (48) under the R environment software 2.14.0 (R Development Core Team 2011). To identify those genes that were significantly associated with high- and low-pH soils, we used Indicator Species Analysis (49) as implemented within the R library labdsv (http://ecology.msu.montana.edu/labdsv/R). The IndVal score for each gene is the product of the relative frequency and relative average abundance within each soil pH group, and significance was calculated through random reassignment of groups (1,000 permutations [Data Set S1]). Networks were plotted using qgraph (http://sachaepskamp.com/qgraph) following construction of a Pearson correlation matrix of all gene abundances and retaining only correlations greater than 0.9. A circular plot showing significant indicator genes, their hierarchical classification within SEED subsystems, and mean relative abundance within pH groups was constructed using the output of the IndVal analyses described above within the R library circlize (https://github.com/jokergoo/circlize).

### Accession number(s).

Raw metagenomic sequences for samples 4475877, 4475885, 4475892, 4475897, 4475881, 4475888, 4475891, and 4475893 are available in the European Nucleotide Archive (ENA) under respective accession numbers ERS078132, ERS078133, ERS078134, ERS078135, ERS078136, ERS078137, ERS078138, and ERS078139. Additional annotated data are available freely at the EBI Metagenomics website (https://www.ebi.ac.uk/metagenomics) under project ERP001068, and by request on the MG-RAST server (http://metagenomics.anl.gov/).
